# Pediatric outpatient parenteral antibiotic therapy: an important option for pediatric hospitals in Germany

**DOI:** 10.3205/dgkh000670

**Published:** 2026-09-01

**Authors:** Jennifer Neubert, Yeliz Akarsu, Rachel Manier, Ulrich von Both, Miriam Stegemann, Arne Simon, TeleKasper Project

**Affiliations:** 1 Praxis für Kinder- und Jugendmedizin, KIJU Praxis Neuss, Neuss, Germany; 2 Pediatric Oncology and Hematology Children’s Hospital Medical Center University Clinics, Homburg, Germany; 3 Pädiatrische Infektiologie, Universitätskinderklinik, LMU Klinikum, von Haunersches Kinderspital München, Munich, Germany; 4 DTMH Standortleitung Infektiologie Campus Virchow-Klinikum, Klinik für Infektiologie und Intensivmedizin, Campus Charité Mitte (CCM), Berlin, Germany

**Keywords:** outpatient parenteral antibiotic therapy, children, oral sequential therapy, antimicrobial stewartship

## Abstract

Outpatient parenteral antibiotic therapy (OPAT) has been established for decades in children and adolescents with cystic fibrosis. Today, prolonged intravenous antibiotic treatment is less frequently required due to the increased use of oral sequential therapy with easily absorbable antibiotics. However, when it is needed in special situations, pediatric OPAT (pOPAT) can be a beneficial and safe alternative for various reasons (especially quality of life, limited inpatient resources, treatment costs). This article takes a critical look at the current status of OPAT in children and adolescents in Germany in order to foster the integration of pOPAT into the overall concept of pediatric infectiology.

## Abstract (de)

Die ambulante parenterale Antibiotikatherapie (APAT) ist bei Kindern und Jugendlichen mit Cystischer Fibrose seit Jahrzehnten etabliert. Heutzutage ist durch den vermehrten Einsatz einer oralen Sequenztherapie mit gut resorbierbaren Antibiotika eine prolongierte intravenöse Behandlung seltener erforderlich. Wenn sie in besonderen Situationen jedoch gebraucht wird, kann die pädiatrische APAT aus verschiedenen Gründen (v.a. Lebensqualität, begrenzte stationäre Ressourcen, Behandlungskosten) eine nützliche und sichere Alternative darstellen. In diesem Beitrag wird ein kritischer Blick auf den aktuellen Stellenwert der APAT bei Kindern und Jugendlichen (pAPAT) in Deutschland geworfen, um diese zu fördern und im Gesamtkonzept der pädiatrischen Infektiologie besser einzubinden.

## Introduction

Outpatient parenteral antibiotic therapy (OPAT) has been known for several decades but its use in Germany has been marginal and limited to the treatment of complicated infections requiring prolonged intravenous antibiotic therapy (ABT). In OPAT, children and adolescents are receiving antibiotics not in the inpatient but in the outpatient setting, i.e. in their home environment or in a paediatrician’s office. The aim is to provide relief to the family and prioritize the patient’s quality of life (without accepting an unacceptable risk of complications).

OPAT also relieves inpatient treatment units, which in some regions can hardly meet the demand for hospital beds in acute care due to a shortage of qualified nursing staff. Of course, qualified nursing staff is also needed for paediatric OPAT (pOPAT), but the well-planned working hours during the day in an outpatient care environment make this option particularly attractive.

This article, based on a narrative review of the available literature concerning pOPAT, represents a consented addendum to the German Guideline S1-Leitlinie “Ambulante parenterale Antiinfektivatherapie (APAT)” of the AWMF (Working Group of Scientific Medical Societies), which has been release online on July 09, 2024 [1]. 

## Primacy of IV therapy?

Many paediatricians and adolescent-medicine specialists believe that intravenous (IV) ABT is safer and, above all, more effective than oral therapy in achieving the therapeutic goal [2], [3], [4]. This assessment is indeed accurate according to current knowledge of initial therapy for severe systemic infections, where the antibiotic concentration in systemic circulation must be particularly high to achieve sufficient exposure at the site of infection, especially in cases with a high pathogen load or difficult-to-reach compartments (e.g., CNS, bones, intraperitoneal or endocarditic vegetations). Prioritizing IV treatment also applies to anti-infectives with poor oral bioavailability (e.g., ampicillin, flucloxacillin, cefuroxime) or patients who, for various reasons, cannot take (or absorb) oral antibiotics.

## Prolonged intravenous therapy: inpatient or outpatient

Some infections require prolonged intravenous ABT. If such treatment is necessary due to the lack of an alternative oral sequential therapy, ABT can be administered either in the hospital or as outpatient intravenous therapy, depending on a number of criteria (see below). OPAT is defined as parenteral (in nearly all cases intravenous) ABT without hospitalization [5], [6], [7], [8]. When it involves children and adolescents, it is referred to as paediatric OPAT (pOPAT). The following treatment situations are distinguished in this context [8]:

*First-line pOPAT (without full inpatient admission)*

- *in the emergency department (or day clinic, or outpatient clinic) of a hospital *[9]
- *in the offices of specialist physicians in private practice*
- *in the home environment (Hospital at Home program) *[8]*, *[10]*, *[11]*, *[12]*, *[13]

*Second-line pOPAT*

- *to shorten a hospital stay under the three above-mentioned structural-organizational/constructio* *n-fu* *nc* *tion* *al framework conditions*

On purely quantitative terms, first-line pOPAT offers by far the greatest potential for reducing inpatient days [8], [14], [15], [16].

## History of OPAT, significance of formal OPAT structures and Antibiotic Stewardship (ABS)

OPAT was first used in 1974 in children with cystic fibrosis [17] and is standard of care in the USA and several other countries (including the UK, Australia, Canada, Brazil) [18], [19], [20], [21]. In the United Kingdom, recommendations from professional societies regarding OPAT for adults and children were first published in 2012 and last revised in 2019 [6]. In practical implementation, there are significant regional variations depending on healthcare structures and available resources [22]. 

An ongoing focus on ensuring patient safety in pOPAT programs has highlighted the importance of a formally defined structure of standardized processes [6], [23]. In recent years, well-established pOPAT programs have also integrated aspects of Antibiotic Stewardship at key control points [24].

## Objectives of pOPAT

The objectives of pOPAT are:

- provision of treatment that complies with guidelines regarding indication, antibiotic choice, dosage, duration, and regimen of administration, ensuring effectiveness and safety of the chosen treatment [24], [25], [26], [27];
- avoidance or reduction of hospitalization (also aiming for a minimal rate of secondary admissions if pOPAT fails);
- reduction of the risk of nosocomial infections associated with hospital stays (especially nosocomial respiratory and gastrointestinal virus infections);
- improvement of patients' quality of life and that of close contacts in their home environments through medical treatment in day clinics, practices, or entirely at home [28], [29], [30], [31], [32], [33];
- reduction of burden on caregiving family members who are concurrently employed part-time or full-time, eliminating the need to accompany their child to the hospital and potentially allowing them to care for other children at home;
- relief of paediatric hospital departments amidst an ongoing shortage of specialized nursing staff and subsequent bed closures. This includes maintaining emergency capacities for treating critically ill children and adolescents, as well as those definitively requiring inpatient treatment (e.g., surgeries, chemotherapy);
- cost savings across various levels.

pOPAT is generally indicated only when no oral treatment alternative is available or patients are unable to take such treatment.

## pOPAT in children and adolescents with cystic fibrosis (CF)

Before the introduction of CFTR modulators, ABT played a significant role in the treatment of patients with cystic fibrosis, being administered orally, via inhalation, or intravenously depending on the indication [34], [35]. pOPAT has been established for several decades in patients with CF [17], [36], [37], [38], [39], [40], [41] and is included in the current treatment guidelines of the AWMF [34], [35].

pOPAT allows patients with CF to continue their daily lives with minimal interruption during exacerbations [42]. OPAT has the potential to reduce the transmission risk of key pathogens (e.g., *Pseudomonas aeruginosa*) among cystic fibrosis patients in hospitals [43], [44], reducing the need for single-room accommodation in hospitals (“CF rooms”) [43], [44]. The advantages of intravenously administered ABT in a hospital setting include better patient monitoring (especially in cases of advanced lung disease) and intensified physiotherapy/inhalation therapy. Data on the safety and effectiveness of OPAT and pOPAT compared to inpatient intravenous therapy are limited (no randomized studies) and show conflicting results [39], [41], [45], [46]. However, randomized studies would be challenging to conduct because patients and their families predominantly prefer the pOPAT option when available [39]. Such a study would also need to ensure comparability with other aspects of acute CF exacerbation management (e.g., inhalation therapy, physiotherapy, nutritional counselling, etc.) between the two groups [42]. With careful patient selection and interdisciplinary therapy including home-based interventions (physiotherapy, nutritional counseling), pOPAT is safe and effective. 

For severely ill children (excluding those in palliative care settings) and those whose families face precarious living conditions, intravenous ABT in an inpatient setting should be preferred [37].

The AWMF guideline [34] states: “Patients with severe pulmonary exacerbations should be treated as inpatients. This also applies to patients with a history of medication intolerance, as well as patients requiring further diagnostic measures and therapy adjustments (e.g., management of diabetes mellitus, optimization of nutritional status, ventilation settings, endoscopies). For all patients, especially children, the home and social situation should be critically assessed to ensure that outpatient therapy is effective and safe; in cases of doubt, inpatient admission is recommended much more generously.”

## Infections that can be eligible for pOPAT

### Skin and soft tissue infections

Patients with preseptal cellulitis (a skin and soft tissue infection of the face) or erysipelas can be successfully treated with pOPAT [47], [48] if oral therapy is not possible [49], [50], [51]. Intravenous therapy may be necessary in cases of rapidly progressing findings, reduced general condition, or if symptoms worsen under ABT [52]. The working group led by Leila F. Ibrahim from the Royal Children’s Hospital in Melbourne has developed and validated a simple clinical score to facilitate the decision for or against intravenous ABT; a score of ≥4 suggests intravenous ABT [53].

The same working group conducted the prospective randomized (monocentric and non-blinded) CHOICE study. In this study, the daily administration of ceftriaxone (once 50 mg/kg/day; pOPAT) was compared with the parenteral administration of flucloxacillin (200 mg/kg/day in 4 doses; hospital group) in children over 5 months of age with cellulitis [54], [55], [56], [57], [58]. Randomization was performed in the emergency department aiming to avoid hospitalization as much as possible in the pOPAT group. Children with orbital involvement, immunosuppression, and significant signs of a systemic inflammatory response, suspected fasciitis, or abscesses were excluded (primarily hospitalized and treated intravenously). The primary endpoint was treatment failure after 48 hours, and the rate in the pOPAT group (home treatment; n=93) was not to exceed the hospital therapy group (n=95) by more than 15%. Oral sequential therapy was possible at the discretion of the physician, with cephalexin (100 mg/kg/day in 4 doses) as the standard antibiotic. The total duration of antibiotic therapy was the same in both groups and relatively long [59] (8.1 days pOPAT vs. 8.3 days hospital), and the mean duration of intravenous therapy differed by only 0.5 days (2.2 days pOPAT vs. 1.7 days hospital group; P=0.045). Treatment failures in the first 48 hours occurred more frequently in the hospital group (7% vs. 2%; risk difference –5.2%; CI 95% –11.3 to 0.8, p=0.088). No advantage could be demonstrated in the pOPAT group regarding recolonization with MRSA, MRGN, or C. difficile (up to 3 months later) [57].

Unfortunately, the study compared two different antibiotic treatment regimens. Due to the significantly narrower antimicrobial spectrum of flucloxacillin (effective against *S. aureus* and usually also against GAS), an intravenous therapy with ampicillin/sulbactam, cefuroxime, or even ceftriaxone might have been more appropriate in the hospital group.

The Australian working group also conducted an economic analysis of their results, finding that the median cost per case in the pOPAT group was 1,965 AUD versus 3,775 AUD in the hospital group (p<0.0001). For 89 children in the pOPAT group treated per protocol, this amounted to a difference of 161,000 AUD (approximately 97,000 EUR). In summary, the authors recommend pOPAT as the new standard [58]. The small difference in the duration of intravenous antibiotic therapy between the two groups also indicates that a significant portion of children initially treated parenterally would qualify for a rapid oral switch. For 1–2 days, the organizational effort of pOPAT at home seems so great that a single daily dose of ceftriaxone in a day clinic or practice is more appropriate (post-hospital treatment?). 

However, this example also shows that, for practical reasons, ceftriaxone (once daily!) is often used more frequently in pOPAT, though less broad-spectrum antibiotics would also be effective and thus a better choice form an AMS point of view.

### Osteomyelitis, septic arthritis

In children with osteomyelitis (OM) and septic arthritis, there are treatment situations where the localization and extent of OM prevent an early oral switch or require surgical interventions [60]. This can be the case, for example, with spondylodiscitis with paravertebral abscess or sequestration of inflamed bone [61], [62], [63], [64], [65]. Additionally, not all children are able to take the high-dose oral sequential therapy required in such cases consistently. In Patel’s study, 45% (n=58) of the cases were osteoarticular infections, while in Fernández-Polo’s (Barcelona) study, 9.4% of the pOPAT cases were osteoarticular infections [66]. 

The proportion of children with osteomyelitis or septic arthritis requiring continuation of parenteral treatment is unknown. Ceftriaxone is not the first choice for severe infections caused by *S. aureus*. Particularly in the case of spondylodiscitis, several weeks of parenteral therapy may be required. Having the option of pOPAT in this case would be advantageous.

In acute mastoiditis, initial intravenous therapy is the accepted standard. Today, most children with acute mastoiditis are treated conservatively (without surgery) with parenteral antibiotics [67]. Typically, depending on severity, the total duration of therapy ranges from 14 to 21 days. With favourable progress and under medical supervision, pOPAT (e.g., with Ceftriaxone) is a feasible option in acute mastoiditis, provided there are no central nervous system complications [68].

### Complicated, initially severe or very severe, community-acquired pneumonia

Children with complicated pleuropneumonia must be initially hospitalized, treated with intravenous antibiotics, and possibly relieved with pleural puncture or drainage [69], [70], [71], [72]. Most of these children fully recover even after extensive parapneumonic effusion/empyema, but only with prolonged antibiotic therapy lasting between 14 and 28 days.

However, there are valid concerns by German expert groups [69], [70] highlighting that most children with complicated pleuropneumonia, who cannot tolerate oral antibiotic sequential therapy, are often still too ill for outpatient treatment and/or still have pleural drains [71]. It is also unclear how many children would benefit from pOPAT in this situation.

### Complicated pyelonephritis

For children with complicated pyelonephritis without signs of severe systemic infection, pOPAT can be a treatment option without hospitalization [73], [74]. In Fernández-Polo’s study (Barcelona), 7.5% of pOPAT cases involved complicated urinary tract infections [66], whereas among the 130 documented pOPAT cycles in Patel et al., there were no urinary tract infections [16].

### CNS Infections

For children under 8 years old with neuroborreliosis, intravenous therapy with ceftriaxone for 14 days is recommended [75]. For children and adolescents who cannot take oral doxycycline and do not have other reasons for hospitalization, pOPAT is a sensible treatment option.

In some children with meningitis who require prolonged intravenous antibiotic therapy (e.g., meningitis caused by B-streptococcus, *E. coli, Enterobacter* spp.) pOPAT at home has been successful after the children were stabilized and afebrile [74]. Children with brain abscess often recover completely after successful neurosurgical intervention once increased intracerebral pressure has subsided. Nevertheless, they sometimes need to be treated with parenteral antibiotics for 28 days or longer. Guidelines of the European professional societies consider a treatment duration of 4 weeks to be the shorter option in cases of favourable progress [76]. An oral sequential therapy has not been sufficiently studied in neither adults nor children [77].

### Complicated intra-abdominal infections

After adequate removal of the intra-abdominal inflammatory focus (complicated appendicitis), a 5-day ABT is sufficient for children and adolescents in good general condition, provided that oral food intake has been successfully established and no persistent leukocytosis is present [78]. In cases of a complicated clinical course of acute appendicitis in childhood, a prolonged intravenous treatment may be required, which can be administered as pOPAT [79]. 

Usually, this treatment involves Ceftriaxone/Metronidazole or Piperacillin/Tazobactam; however, the latter does not appear to be advantageous without evidence of *P. aeruginosa* (or without immunosuppression) [80]. After a complicated intra-abdominal infection, children require close clinical monitoring (focal or systemic signs of inflammation, constipation, ileus, adequate intake of food and fluids, pain management). Even with prolonged ABT, there remains a significant probability (5–15%) of complications (e.g., secondary abscesses). Therefore, pOPAT does not eliminate the need to examine such a child at least twice a week and as needed clinically (and if necessary, by sonography).

## Fever in neutropenic cancer patients

If appropriate outpatient care structures are available, OPAT is an option for stable paediatric cancer patients with fever during neutropenia [13], as long as they are not at increased risk for complications. However, according to the German AWMF guideline Reg. No. 048/014 (Version 2024), the minimum duration of therapy is only 72 hours (only 48 hours if granulocytes recover earlier) [81], [82] so OPAT might be more suitable for targeted therapy of BSI (with identified pathogens) in this patient population. An example of this is the* in situ* treatment of a CVAD-associated bacteraemia caused by coagulase-negative staphylococci (CoNS) with teicoplanin (once daily from the fourth dose for a total of 7 days) [83], [84] or daptomycin [85], [86], [87], [88]. 

The only prospective randomized study included in the systematic review by Bryant et al. 2018 [89] on OPAT was a study on the treatment of FN in the home setting [90]. 

Haeussler et al. report that patients and their families view early discharge and continuation of parenteral ABT at home as a significant advantage [12] and would generally choose this option again once they have experienced it [28], [29], [91]. The “There is no place like home” pOPAT program in Melbourne [13] was meticulously planned, implemented, and supervised [92]. Treating pediatric oncologists see the clear structural, organizational, and personnel allocation of resources as a prerequisite for allowing children with FN and a low risk of complications (e.g., sterile blood culture after 24 hours, no continuing fever, good general condition, no severe mucositis) to leave the hospital earlier [12]. Interestingly, the Hospital at Home program saw a significant rise during the pandemic due to patients and their families fearing nosocomial SARS-CoV-2 infections. Telemedical interventions can also play a role here. Hospital at home initiatives exist in various pediatric oncology centers for less intensive parenteral chemotherapy (e.g., intravenous bolus doses of vinblastine or cytarabine) [93], [94], [95], [96]. 

This could be relevant for pOPAT due to synergies with the resources allocated for outpatient care. An online discussion between the authors and participants of the DGPI OPAT survey revealed that most clinics currently lack the structural, organizational, and personnel prerequisites for pOPAT in pediatric oncology patients. On the other hand, there are recent reports [97] of promising pilot projects, such as “KIKHomeCare – Outreach outpatient care for children and adolescents with cancer” (http://brueckenteam.org/) in a relatively densely populated region with several pediatric oncology centers (Cologne, Essen, Dortmund, and the pediatric research network).

### Expert consultation

At the beginning of pOPAT and throughout its course, paediatric infectious disease specialists should be consulted. Ideally, they would be members of the pOPAT team [10], [24], [25], [98]. Such consultations can also occur within the framework of cooperation between different paediatric hospitals, for which regional paediatric infectious disease networks provide the structural and organizational framework.

They show that, **before initiating pOPAT, both the indication for continuing ABT and the indication for its parenteral administration should be expertly reviewed** (discharge stewardship) [26], [27]. 

In the study by Xu et al., there was an initial possibility for oral sequential therapy in 24% (75 out of 317) of all index cases, and during follow-up, in 28% (213 out of 749) of subsequent consultations [9]. 

Knackstedt et al. [99] defined eight categories of ABS intervention when reviewing pOPAT cycles:

1. discontinuation of ABT,
2. oral sequential therapy,
3. treatment duration,
4. reduction of individual doses (change in administration schedule),
5. antibiotic switch,
6. termination of combination therapies,
7. dose modification, and
8. laboratory monitoring.

In a retrospective analysis of 401 pOPAT cycles in 301 children and adolescents, 45% had received a pediatric infectious disease consultation. For cycles without a consultation, the ID service would have definitely recommended a change or suggested considering one in 78% of the cases. In 40%, there was either no indication to continue ABT or there was an option for oral sequential therapy.

## Prerequisites for OPAT in children

In principle, it is always advisable to involve a pediatric infectious disease specialist when deciding for or against pOPAT and in detailed planning (selection of the antibiotic, dosage, duration of therapy, etc.). This refers to a physician who holds the additional designation of infectious disease specialist. Clinics that lack this expertise should contact a treatment center with pediatric infectious disease specialization within their region through regional cross-sectoral antimicrobial stewardship networks.

pOPAT is just one potential area for such cooperation, the specifics of which should be outlined in a cooperation agreement. This approach ensures that expertise is brought to the patient.

The inclusion criteria for pOPAT at the University Children’s Hospital in Barcelona [66] are well-suited as a guide, and are briefly outlined as follows:

- There is no suitable oral treatment option for this condition and these patients.
- Patients are clinically stable (suitable for outpatient treatment) and have appropriate vascular access.
- Adult caregivers (home caregivers) feel (and are) capable of performing pOPAT; they can acquire the necessary knowledge/skills in the short term.
- Communication with the family should be possible at all times without specific barriers (accessibility, language).
- Adequate training of patients and their close contacts in the home environment should be ensured.
- Follow-up appointments for clinical monitoring (including blood sampling, if necessary) must be strictly adhered to by the family. Reliability and trust should therefore be established on both sides.

Ideally, a pharmacist should be a member of the pOPAT team. When the antibiotic to be administered during pOPAT is professionally reconstituted by a pharmacy [100], delivery (potentially including the infusion set with a peripheral back-check valve) can be arranged for several consecutive days. The physical-chemical stability of the reconstituted solution is crucial in this process [101], [102], [103], [104]. Subsequently, only flushing the vascular access with sterile saline solution, preferably using pre-packaged sterile saline flush syringes, and changing the infusion bag on-site are required.

We do not consider it appropriate for parents to reconstitute antibiotics themselves before administration because the necessary minimum infection prevention standards cannot be reliably maintained [105], [106].

Obviously, patients should not have any allergies to the antibiotics used in OPAT [107], [108].

In a retrospective study (January 2016 to April 2019), Elizabeth Townsley and her pediatric infectious disease team at Vanderbilt University Medical Center (Nashville, Tennessee) analyzed risk factors for complications during pOPAT based on data from 181 pOPAT cycles. In 39% of cases, an adverse event occurred (significantly less frequently in rural compared to urban regions). Medication errors (e.g., incorrect dosage) accounted for 16.6% of these incidents. Problems with vascular access occurred in 26% of all cycles. Each additional day of pOPAT increased the risk of vascular catheter complications by 4%. Complications necessitating hospital admission occurred in only 20 out of 181 cycles (11%) [109].

Regarding individual family circumstances, the decision for or against pOPAT can be complex, with a risk of discrimination against children from socioeconomically disadvantaged families, who may not be offered pOPAT [110]. The success of this approach hinges on the actual presence and tested efficacy of safety nets, particularly related to the conditions of the outpatient care structure [111].

### Selection of antibiotics for OPAT in children

For a pOPAT regimen to be successful, the antibiotic should be effective and safe. Additionally, administration should be straightforward and practical for everyday use.

Before prescribing an antibiotic for pOPAT, the following questions should be addressed:

- What vascular access is available (e.g., MLC, PICC, CVAD)?
- Where will the pOPAT be administered (e.g., day clinic, practice, home therapy)?
- Who will administer the antibiotic during home therapy (family member, caregiver, health care worker), and how frequently must the antibiotic be administered?
- Who will perform quality- & maintenance care of the vascular access (flushing, aseptic dressing changes), and who will train the family members if necessary? Are there training materials available (handouts)?
- What method of administration will be used (gravity-fed short infusion, IV bolus, elastomeric pump, Perfusor™)?
- What costs will be covered by health insurance (is approval for pOPAT granted)?
- What are the pharmaceutical properties, especially the physical-chemical stability after reconstitution and at room temperature?
- What side effects can be expected, and what monitoring is necessary?
- Are there potential interactions with other medications taken by the patient?

Particularly when antibiotics are delivered and stored in the patient’s home, the stability of the drug after reconstitution is critical. In a day clinic, reconstitution is usually performed immediately before administering the drug by medical professionals, and this typically involves short infusions. If reconstitution is not done in a pharmacy under cleanroom conditions as per the professional information, the ABT must be connected to the vascular access within one hour (“one-hour rule”). However, if a licensed pharmacy provides the ABT, pharmacists can determine the shelf life; in this case, the one-hour rule does not apply. Stability data after reconstitution for pharmacists can be found partly in the professional information and in the Extended Stability for Parenteral Drugs handbook by the American Society of Health-System Pharmacists (7^th^ edition, August 2022). The updated UK guidelines (2019) specifically highlight the lack of stability data for many antibiotics, including in elastomeric pumps [6]. The British Society for Antimicrobial Chemotherapy operates its own Drug Stability Testing Program to generate reliable data on the stability of antibiotics in the OPAT setting.

The IDSA Guidelines and the e-handbook on OPAT also provide detailed information on the antibiotics commonly used in OPAT (Norris et al. CID 2019, Handbook of Outpatient Parenteral Antimicrobial Therapy for Infectious Diseases, 3^rd^ Edition 2016). Since 2019, several excellent overviews on this topic have been published [101], [102], [103], [104], [112].

Antibiotics with long physical-chemical stability (24 hours at room temperature) include Benzylpenicillin G, Flucloxacillin, Piperacillin/Tazobactam, Cefazolin, Cefepime, and Ceftaroline [102], [103], [113].

Medications such as Ampicillin, Ampicillin/Sulbactam, Meropenem, or Imipenem/Cilastin exhibit only limited stability at room temperature and are therefore poorly suited for continuous infusions. These antibiotics need to be administered multiple times a day. The stability of the antibiotic significantly depends on the solvent and the application system, as well as the ambient temperature (close to the body vs. away from the body). For example, the stability of Meropenem in an elastomeric pump is limited [114]. For children, programmable pumps for multiple daily administrations are more compatible with daily life than continuous infusions. However, there are also safe infusion pumps for the latter, where the antibiotic is delivered in a bag that is carried along with the infusion pump in a backpack. Such applications are used in pediatric oncology for therapeutic monoclonal antibodies that are administered as continuous infusions over days to weeks (e.g., Blinatumomab, Dinutuximab). These pumps can be rented from a central distributor, so technical issues are primarily handled through an emergency phone line provided by the company.

The Good Practice Recommendations for Paediatric Outpatient Parenteral Therapy from 2015 and the update from 2019 list the antibiotics most commonly used in OPAT, along with their indications, methods of administration (infusion duration, frequency, etc.), potential side effects, and necessary monitoring [6], [7].

## Monitoring of laboratory values during pOPAT

Systematic prospective studies on the benefits of laboratory monitoring during pOPAT are not available. Nevertheless, laboratory tests are recommended, particularly:

- in cases of impaired kidney function, and when fluctuations of renal function are expected;
- when the antibiotic used is likely to cause adverse effects of body functions and thus monitoring of laboratory parameters is warranted (such as leukocyte or neutrophikl counts, liver enzymes, serum creatinine levels).

In the 2018 study by Patel et al. [16], tests were conducted at least weekly, including a complete blood count with differential, liver and kidney function tests, C-reactive protein, and creatine kinase in children and adolescents treated with daptomycin (n=5; 3.8%). For these tests, patients had to visit the responsible pediatric clinic on an outpatient basis once a week.

## Specific considerations for children

Children and adolescents typically cannot administer parenteral antibiotics themselves. The general condition of a child receiving OPAT and the state of the vascular access must be competently monitored. To a certain extend, this can be managed by adequately instructed (and practically trained) adult caregivers living in the same household. 

However, even adult caregivers (parents, guardians) are not always able to acquire the necessary knowledge and skills in a short period of time. 

Not all parents feel comfortable taking on more complex medical tasks in monitoring and caring for their child [66], [115], and pOPAT should not overwhelm the family. In the UK, there are pediatric community nurses who collaborate closely with the relevant pediatric departments and can be involved in monitoring pOPAT [16]. Similar structures are also part of the Australian pOPAT program [92], [116]. In the KIKHomeCare pilot project, oncology-trained nursing staff provide home care [97].

The side-effect profile of ABT can differ between children and adults. For example, blood sampling to monitor blood counts and other laboratory values [117] can be more challenging and may require more personnel in children compared to adults. 

Similarly, the same applies to the higher effort involved when a peripheral venous catheter (PVC) or another vascular access needs to be newly inserted in a child [118], [119], [120], [121], [122]. On the other hand, children often have fewer comorbidities (kidney function, liver function) and accompanying medications, which reduces the likelihood of adverse effects from ABT. 

In children, the most commonly used antibiotics for pOPAT include Piperacillin/Tazobactam, Ceftriaxone, Ceftazidime, Meropenem, and Teicoplanin, with this distribution significantly influenced by the proportion of patients undergoing pOPAT for cystic fibrosis [66]. 

In the study by Patel et al., Ceftriaxone predominated (79%), while Piperacillin/Tazobactam, Flucloxacillin, Teicoplanin, and Daptomycin were used less frequently.

Delayed antibiotic adverse reactions (e.g., rash, leukopenia, liver enzyme elevations) have been reported in children undergoing pOPAT, but with appropriate monitoring, these are not arguments against its use [16].

In the study by Fernández-Polo et al. (Barcelona) [66] pOPAT was primarily successful in 75.4% of cases (n=79 cycles). In the remaining 27 cases (25.5%), it was either switched prematurely or discontinued in favor of inpatient therapy. Among these 27 cycles (100%), the reasons leading to discontinuation of pOPAT included inadequate infection response in 37%, the need for adjustment of antibiotic therapy for optimization in 29.6%, catheter occlusions in 22%, and adverse effects in 11%. In the study by Patel et al., the proportion of unsuccessful or prematurely terminated pOPAT cycles was 3.9% (5 out of 130).

### Proactively limit the duration of therapy

The pOPAT must not prolong the duration of therapy for structural or organizational reasons (e.g., waiting until the next scheduled outpatient appointment) [16]. It is an important task of the accompanying AMS team/pediatric infectious disease specialists to proactively limit the duration of therapy. To avoid jeopardizing individual patients with an undifferentiated stopping rule, each pOPAT should conclude with a clinical cessation consultation.

## Vascular catheters for pOPAT in children

Although there is no longer a fixed time interval for changing a functioning peripheral venous catheter (PVC) with a painless insertion site in children today [123], a PVC inserted in mobile (non-sedated) children frequently does not last for more than 2 to 3 days [124]. 

This holds true even in departments where a standardized maintenance care protocol is implemented [125]. Therefore, PVCs are generally not suitable vascular accesses for pOPAT, which typically lasts at least 4 days. 

As a practical alternative with a lower risk of phlebitis or dislocation, midline catheters (MLCs) have been established [119], [126]. These are 5–10 cm long vascular catheters, typically inserted via the Seldinger technique into the brachiocephalic vein and advanced so that the tip lies near the junction with the axillary vein. Unlike central venous catheters (CVCs), a chest X-ray is not required to confirm catheter placement. However, MLCs should not be used for infusions requiring central catheter tip placement, such as total parenteral nutrition. In a randomized study involving 127 patients, MLCs showed lower complication and replacement rates compared to PVCs and had a significantly longer dwell time (median 66.9 hours; P<.001). This resulted in higher patient and parent satisfaction with treatment, along with lower overall costs in the MLC group [126]. Studies by other research groups have demonstrated similar outcomes [118], [127]. Often, an appropriate vein is identified using ultrasound or a vein scanner before puncturing to place a MLC [126], [128]. However, a MLC can also lead to venous thrombosis [127]. One group utilizes adult arterial catheters as MLCs in children [118]. 

Fläring et al. recently published a retrospective analysis of monocentric data on the use of MLCs in the context of pOPAT at the Karolinska University Hospital in Stockholm [129]. A total of 41 MLC placements in children with a mean age of 5.9 years were included. Twenty percent of the patients were younger than 12 months. Placement was performed by pediatric anesthesiologists. In 76% of cases, pOPAT was successfully administered exclusively at home by community-based nursing staff, overseen by a designated ‘Hospital in the Home physician’. 

The median duration of pOPAT was 7 (5–10) days, with Ceftriaxone being the most frequently prescribed antibiotic. MLC-related complications occurred in 34% (n=14) of cases, with insertion site pain being the most common (24%), leading to catheter removal. There was one MLC-associated venous thrombosis (2.4%). The authors consider the use of MLCs a viable option in the context of pOPAT, noting that the saphenous vein may be less suitable as an insertion site.

Another option is the peripherally inserted central catheter (PICC), which is inserted through a peripheral vein but with its tip in a central vein [130]. The PICC is a central venous catheter, and strict aseptic precautions should be observed during insertion [106], [131].

In a comparative study from 2007, the ‘survival time’ of CVCs and PICCs far exceeded the duration typically required for OPAT (61 days for CVCs and 41 days for PICCs) [122].

At Fernández-Polo’s clinic in Barcelona, most children undergoing OPAT had a MLC or a PICC [66]. In Patel et al.’s study [16], there were 10 children and adolescents with Broviac catheters (7.6%), while most others received OPAT via a PICC (81%); PVCs were rarely used (11.5%).

Wang et al. [130] examined the complication rates of PICCs and indicated that uncomplicated insertion and absence of bleeding from the insertion site immediately after placement reduce the risk of complications. Kleidon et al. [119] compared PICCs and midline catheters (MICLs) in a randomized study involving 110 children and adolescents (mostly with CF) [119]. MICL placement required anesthesia less frequently (10% vs. 69%). There was a higher incidence of MICLs requiring replacement compared to PICCs (18.1 vs. 5.5 events per 1,000 catheter-days); however, this difference appears to have leveled out over the course of the study due to a training effect.

Kovacich et al. [120] from Johns Hopkins Children’s Center investigated the use of PICCs between 2003 and 2013 (14,565 catheters in 955 children and adolescents). 8% of all catheters required early removal or replacement (4.6 events per 1,000 catheter-days). Interestingly, children transferred to nursing facilities had a higher risk of complications. Younger children, those from less socially privileged families (insurance status as a proxy), and those with non-central PICC tip placement also faced higher complication risks. Remarkably, 32% of children with PICC-related complications did not actually require pOPAT after discharge from the hospital. Another U.S. study by van Winkle et al. [121] (2003–2006) described the course of 39 PICCs in 34 children and adolescents undergoing pOPAT at a smaller regional pediatric hospital. The mean catheter dwell time was 20.5 ±13.9 days. 97% of all children successfully completed pOPAT at home, with 82% requiring only one catheter placement. Even then, pOPAT led to a daily cost savings of approximately $1,000 USD [121].

In summary, the available data for pOPAT supports the use of MLCs or PICCs, unless the child already has a permanent implanted central venous access device like a Hickman/Broviac or Port (CVAD). The insertion of MLCs or PICCs should be performed by well-trained medical personnel. Whether analgosedation of the child is necessary to ensure stress-free insertion under optimal aseptic conditions is decided on a case-by-case basis in consultation with the caregivers.

## Results of a survey on the practice of pOPAT in German pediatric clinics

A web-based survey on established pOPAT practices was conducted in October 2023. Participants were invited via the email distribution list of the German Society for Pediatric Infectious Diseases (DGPI). In total, employees from 45 paediatric hospitals participated, including 5 specialists, 27 senior physicians, and 13 chief physicians.

67% (30/45) of the clinics have a pediatric ABS team, with 63% (19/30) having at least one on-site physician with additional qualifications in clinical infectiology, and 61% (n=27/44; the question was skipped by 1 participant) having pharmacists with specialized knowledge and experience in pediatric pharmaceutical issues (responsible for the pediatric clinic). 

Only 29% (n=13/45) fulfill all three structural features for an adequately staffed pediatric ABS team. Half of the respondents (n=22/44) indicated that their clinics offer pOPAT.

Most pOPATs were administered at home (n=19/22, 86%) or in a day clinic (n=13/22, 59%). The most common indications for pOPAT were antibiotic therapy for patients with cystic fibrosis, neuroborreliosis, and osteomyelitis (Table 1 (Tab. 1)).

In the survey, participants from hospitals without established pOPAT were asked whether they generally consider it useful to establish and create the structural, organizational, and personnel prerequisites for pOPAT so that children can continue antibiotic treatment in an outpatient setting, if necessary. Participants from a total of n=22 hospitals responded as follows: n=9/22 (40.9%) fully agree, n=11/22 (50.0%) agree, n=1/22 (4.6%) neither agree nor disagree, and n=1/22 (4.6%) disagree. Nineteen out of 22 clinics with an established pOPAT program expressed interest in participating in an online exchange on this topic.

## Supplementary notes on oral sequential therapy in childhood

For many infections, adequately dosed oral antibiotic therapy with an appropriate antibiotic is not inferior to intravenous treatment [132]; therefore, oral treatment can be initiated a priori or switched to oral sequential therapy as early as possible [133], [134]. Oral sequential therapy can shorten the duration of hospital stay, improve quality of life for patients and their families, reduce the risk of nosocomial infections, and significantly lower treatment costs. The potential of oral sequential therapy is likely not fully utilized [135].

Early oral switch in children with osteomyelitis (OM) and septic arthritis (SA) showing favorable outcomes under initial parenteral antibiotic therapy (uncomplicated OM or SA) has been investigated in scientific studies over the past 10 years and has proven effective [136], [137], [138], [139], [140], [141]. 

A prerequisite is defervescence, a significant reduction in pain, and (if initially elevated) a halving of the CRP value or a decrease below 20 mg/L [142], [143]. Although this has been demonstrated primarily for MSSA, it also applies to uncomplicated cases of MRSA; Clindamycin is an appropriate oral antibiotic for this group of patients with community-acquired MRSA (caMRSA).

In cases of acute mastoiditis without CNS complications, cohort studies have shown successful treatment of children following surgical intervention with exclusively orally administered sequential therapy [144]. Therefore, in most cases with a favorable outcome, an oral switch is possible after 7 days [144].

In many cases of complicated community-acquired pneumonia with pleural empyema, it is possible to continue targeted oral treatment in children once the pathogen is identified, the pleural empyema no longer requires drainage, and they have responded well to initial parenteral antibiotics [145], [146], [147]. In Shah et al., the median hospital stay in both groups (oral vs. pOPAT) was 7 and 9 days, respectively, which is significantly shorter than the median hospital stay reported in a registry study from Germany [17 days (IQR 13–24 days)] [70]. Patients received antibiotics for an additional 14 days, with only 5% requiring continued treatment for 21 days or more after discharge.

In pediatric complicated pyelonephritis, the duration of IV antibiotic therapy is not decisive for treatment success [148], [149]. This applies even if the pathogen was initially detected in the blood culture as well [149], [150]. When the pathogen is known, targeted oral antibiotic therapy is possible in most cases. Whether early oral sequential therapy is also a safe treatment strategy for a renal abscess (originating from pyelonephritis) or a renal carbuncle (following hematogenous septic spread) has not been specifically studied under controlled conditions. The AWMF (Association of the Scientific Medical Societies in Germany) guideline on urinary tract infections in childhood recommends a “three-week, parenterally initiated antibacterial therapy with an antibiotic tested as sensitive” in these cases [151].

In children aged 8 years and above, oral doxycycline (duration of therapy 14–21 days) is the treatment of choice for neuroborreliosis or Lyme arthritis [75].

If patients with perforated appendicitis are able to take oral antibiotics, oral sequential therapy (e.g., with amoxicillin-clavulanate after 3 days of IV therapy) is as effective and safe as IV therapy (in the hospital or as pOPAT) [79], [152], [153]. Complications associated with the use of a vascular catheter are eliminated with oral therapy [154].

Controlled, evidence-based studies on the optimal duration of therapy for bloodstream infections (BSI) in children and adolescents are largely lacking [139]. However, there is no reason to assume that outcomes in children are more complicated than those in adults. Therefore, for uncomplicated BSI, a shortened antibiotic therapy duration of 7 days is indicated [155]. (For *Staphylococcus* *aureus* infections, a minimum of 14 days is still recommended, with discussions ongoing regarding the optimal timing to transition to oral sequential therapy) [156], [157].

## Notes

### Authors’ ORCIDs

- Neubert J: https://orcid.org/0009-0000-2456-225X
- Akarsu Y: https://orcid.org/0009-0009-4057-562X
- ManierR: https://orcid.org/0000-0003-0562-2901
- von Both U: https://orcid.org/0000-0001-8411-1071
- Stegemann M: https://orcid.org/0000-0002-7968-0429
- Simon A: https://orcid.org/0000-0001-9558-3330

### Funding 

None. 

### Acknowledgments

We would like to thank the working group of the AWMF guideline on OPAT for their collaboration, and Janina Soler Wenglein, Markus Knuf, Johannes Forster, Luise Martin, Tobias Tenenbaum, and Nicole Töpfner from the DGPI board for critically reviewing the manuscript draft.

### Competing interests

Arne Simon and Ulrich von Both are members of the Antibiotic Stewardship Working Group of the DGPI and the extended DGPI board. Both, along with Jennifer Neubert, are members of the Working Group on Outpatient Pediatric Antibiotic Stewardship of the DGPI. Miriam Stegemann coordinates the Working Group of the OPAT Guideline of the S1 Guideline Registry Number 092-004 “Ambulatory Parenteral Antimicrobial Therapy (OPAT)”. Yeliz Akarsu, Rachel Müller, Arne Simon, and Ulrich von Both are researchers in the TeleKasper Project (GBA Innovation Committee Funding Code 01NVF19009). Ulrich von Both is a member of the ESPID/ESCMID pediatric antimicrobial stewardship Network.

## Figures and Tables

**Table 1:**
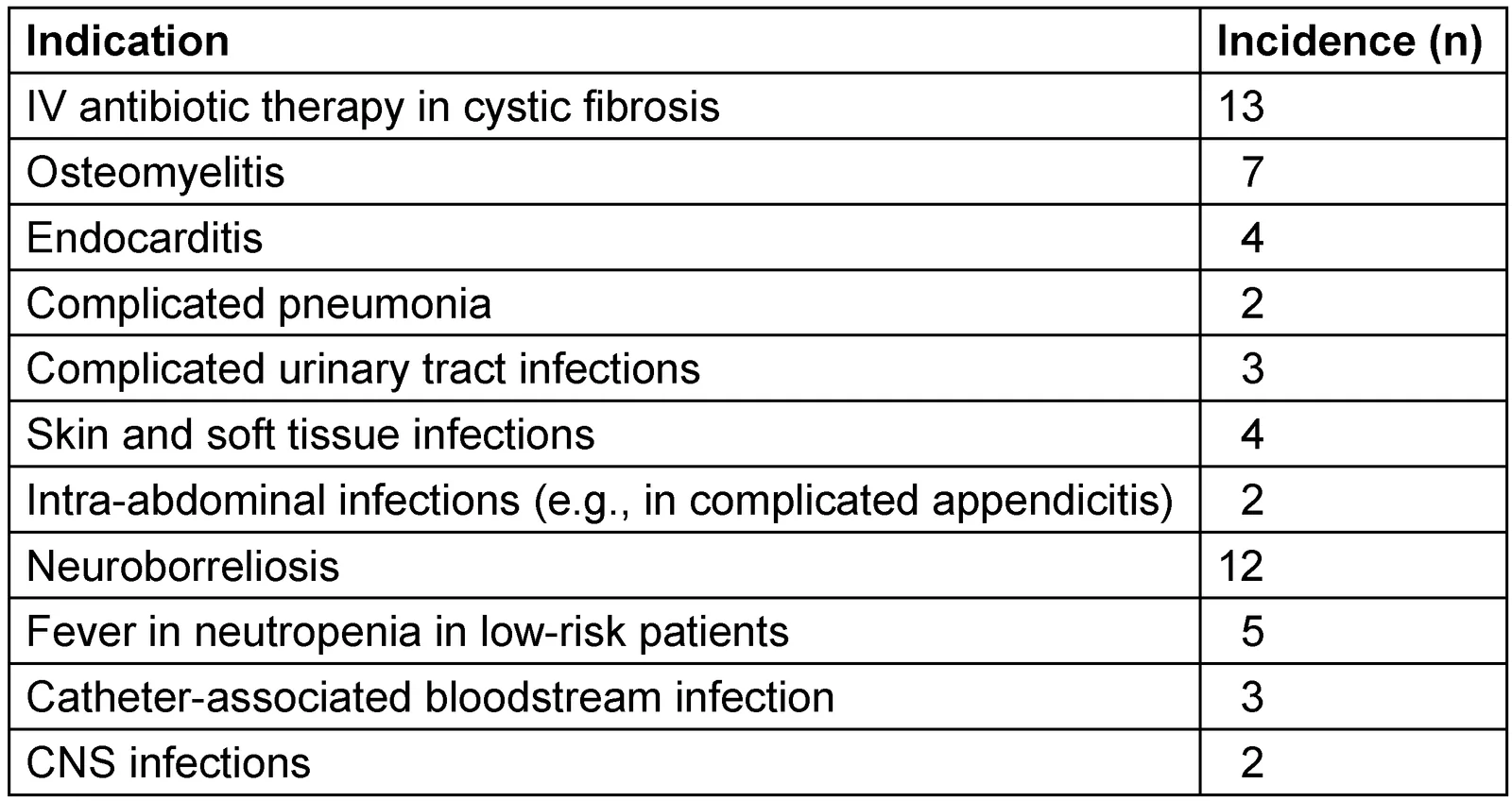
Most common indications for pOPAT in the pOPAT survey of the DGPI (2023)
